# The Past and Future in Dentistry

**Published:** 1902-06-15

**Authors:** F. H. Essig


### THE PAST AND FUTURE IN DENTISTRY.

BY F. H. ESSIG, D.D.S.

Read before the Southwestern Michigan Dental Society, April 8, 1902.

I shall not endeavor to present to you to-day a lengthy paper giving you a treatise in detail, for your consideration, but shall only try to present a few things that serve as an incentive to further discussion.

I am inclined to hesitate, feeling that my experience had been too limited, but gazing at my diploma, I found that fourteen years had elapsed since I was allowed to practice my profession. In this brief time, as it appears to me, I look back and note many changes, a few of which I shall mention.

The first that comes to my mind is the wonderful improvement in facilities that go to make the practice of dentistry better and easier.

If we were to recall the equipment of the dentist of twenty years ago, we should find it far different from that of one of to-day. I need not describe that office as the most of you are in possession of the facts, but when we step into the offices of to-day, with their beautiful chairs, fountain cuspidors, electric lathes and engines, seamless crown systems, we take off our hats in amazement and wonder how our predecessors did their work.

To my mind, that which has come under my special notice during the past fourteen years and has made more advancement than any one thing in dentistry, is the art of crown and bridge-work. Then this branch of dentistry was not universally adopted by the profession, and some of our best operators discouraged it, while others could not sing its praises too high. At that time it was more of a specialty, in fact but few were using bridge-work.

I was taught in my college course to look upon it with suspicion and was even told by some of my instructors not to use it. In my first year's practice I certainly did discourage it, but I soon found that it was necessary in order to keep up with my confreres, that I should have to adopt it. In Dowagiac I found a specialist in this work as my competitor. I immediately began to make a study of it and I well remember my first experience at it. I have watched it from that time until to-day we see it almost universally adopted. I am willing to believe that many a tooth has been saved by the crown, and many a plate discarded by the use of a suitable bridge. To be sure we are not all experts but we do the best we can, and we shall hope to improve by experience until we become more proficient in this art.

The next advancement is the number of teeth that are saved by careful treatment compared with twenty years ago. We are able to-day to save almost any tooth by the use of the remedies that are at our command, the new light on disease, and a more accurate knowledge of how to use the remedies at our command. The almost endless list of disinfectant and antiseptic drugs, and the local anesthetics and anodynes, serve to illustrate this statement.

We have come to see the tortures of the dental chair lessened by the newer modes of treatment, and I hope that we may still advance until the dread of to-day may become the pleasure and duty of to-morrow.

Orthodontia has made such progress that to-day we are able to transform an irregular shaped mouth to one of beauty and usefulness. There is a broad field for this kind of practice, but I believe it should be more of a specialty, not universally practiced. as the average dentist is too busy to devote the proper time to it.

In oral surgery we notice a great advancement made in the past few years by specialists aided by the X-ray and other wonderful inventions until lives have been saved and many a home made brighter.

The special education of the public as to the care of the teeth has been greatly neglected, and it is along this line that we have made the least advancement, the reasons for which are many. There is hardly a day that we are not called upon to remove the first permanent molar tooth, the parent informing us that it is only a "baby tooth. Most of the people are unable to tell us but little about the teeth, have very slight conception of physical or functional characteristics of the teeth, due no doubt, to a lack of educational effort by the dentist, and proper popular dental literature. The average dentist is too busy to deliver a lecture of from fifteen to twenty minutes to each individual that comes to him for a dental operation, and I doubt if a majority of the people would care to listen to it. The question then arises: How shall we reach this matter intelligently and successfully? It seems to me we are held back by selfishness or the fear of committing a breach of our ethical code.

To my mind the proper way to get at this matter is through the public school system, dental periodicals, or popular literature placed easily accessible to the people. I am glad that the National Dental Association has taken this work in hand, and I am hopeful that in the future good things for humanity as well as for our profession will be attained. The field for the future is a broad one, inviting and urging us to press on to make even greater progress than we have in the past. Let us be progressive, ready to accept and work out every good thing, until the people will not dread and put off the care of their teeth on account of the fear of a dreadful dental operation.

